# Isolated Chronic Neutropenia in Adults: Causes, Diagnostic Work-Up, and Management—A Narrative Review

**DOI:** 10.3390/jcm14217495

**Published:** 2025-10-23

**Authors:** Linet Njue, Naomi Porret, Martin Andres, Alicia Rovó

**Affiliations:** 1 Department of Hematology and Central Hematology Laboratory, Inselspital, Bern University Hospital, 3010 Bern, Switzerland; naomiazur.porret@insel.ch (N.P.); martin.andres@insel.ch (M.A.); alicia.rovo@insel.ch (A.R.); 2 Department for BioMedical Research, University of Bern, 3008 Bern, Switzerland

**Keywords:** chronic neutropenia, acquired, congenital, infections, granulocyte colony-stimulating factor

## Abstract

Neutropenia is certainly of clinical significance due to its increased risk of infections in most patients. Chronic neutropenia is defined as neutropenia that persists for more than 3 months. Isolated chronic neutropenia is rare in clinical practice, and its differential diagnosis can be challenging. This rare entity is the focus of this review. Here, we examine the common causes (drugs, hereditary, autoimmune, and idiopathic), diagnostic work-up, and management of chronic isolated neutropenia in adults. We also discuss the Duffy-null-associated neutrophil count (DANC), which has a high prevalence (80–100%) in Sub-Saharan Africans and in Arabs and is not considered a medical condition. It should be highlighted that management decisions in patients with chronic isolated neutropenia should be individualized, mainly taking into account their clinical history over the neutrophil count alone. In this narrative review, we exclusively focus on non-malignant, non-cytotoxic and non-chemotherapy-induced forms of isolated chronic neutropenia in adults.

## 1. Introduction: Definition and Classification

Neutropenia is characterized by an absolute neutrophil count (ANC) below 1.8 × 10^9^/L in adults [1,2]. Neutropenia can be classified according to severity, disease course, and etiology. Classification by severity comprises three categories: mild (ANC 1.0–<1.8 × 10^9^/L), moderate (ANC 0.5–<1.0 × 10^9^/L), and severe (ANC < 0.5 × 10^9^/L) [2]. The term agranulocytosis refers to an ANC < 0.2 × 10^9^/L, and is usually associated with fever. Normal reference ranges, however, differ by ancestry due to the Duffy-null phenotype, as discussed below. It is also important to note that only mature granulocytes (band and segmented granulocytes) are taken into account in the calculation of the ANC. Severe isolated neutropenia is rare [3].

Depending on its duration, neutropenia can be classified as either acute or chronic [4]. While acute neutropenia evolves over a few days or weeks, chronic neutropenia persists for 3 months or longer (Table 1). Drugs and toxins are the primary causes of acute neutropenia in adults [5]. Febrile neutropenia is a medical emergency that can be caused by numerous drugs, including antineoplastic therapies, and often requires hospitalization and broad-spectrum antibiotics [6].

Etiologies of isolated neutropenia are heterogeneous; it may accompany a wide range of conditions, from benign variants, transient viral diseases, or exposure to certain drugs, to potentially life-threatening disorders. Furthermore, isolated neutropenia could have malignant or non-malignant causes.

In this review, we focus on non-malignant and non-chemotherapy-induced forms of isolated chronic neutropenia in adults. Neutropenia caused by chimeric antigen receptor (CAR) T-cell therapy or bispecific antibodies is also excluded in this review.

## 2. Diagnostic Work-Up

The initial steps in the diagnostic assessment of patients with isolated neutropenia should include a thorough medical and family history. This detailed family history should include ethnic origin and history of neutropenia in other family members. Drug history is especially important for adult patients, along with investigations for infections and autoimmune disorders that may be associated with neutropenia. First-line investigations should include complete blood counts, blood smear, liver and kidney function tests, as well as testing for nutritional deficiencies, immune deficiencies, and rheumatologic disorders. Furthermore, flow cytometry evaluating lymphocytes from peripheral blood samples may contribute to the identification of hematological neoplasms. T-cell large granular lymphocyte leukemia (T-LGLL) as a classic example of a disorder where flow cytometry is crucial for diagnosis [7]. Viral serology screening for “Epstein–Barr virus” (EBV), “Cytomegalovirus” (CMV), “Hepatitis B” and “Hepatitis C” viruses, “parvovirus B19”, and “Human immunodeficiency virus” (HIV) is also recommended [2,7]. According to current guidelines, testing for antineutrophil antibodies is recommended among the first-line investigations of unexplained neutropenia [2]. A positive indirect granulocyte immunofluorescence test (GIFT) should ideally be performed as a first-line test in a reference laboratory and repeated if clinical suspicion of autoimmune neutropenia (AIN) is high. A positive GIFT, together with suggestive clinical features, could support the diagnosis of AIN. A positive test, however, does not exclude other types of neutropenia, and could even be found in the absence of neutropenia [8]. Management strategies should, therefore, not be based on the results of antineutrophil antibody testing alone [4].

Recommended second-line investigations depend on the duration and severity of neutropenia. These include testing for copper levels, serum electrophoresis, and serum complement levels, as well as bone marrow investigations, including next-generation sequencing (NGS) panel tests for myeloid neoplasms [2,4]. Hereditary genetic testing is recommended for patients with chronic isolated neutropenia and positive family history to rule out or confirm congenital neutropenia if the second-line tests are inconclusive [7]. Other rare inherited marrow failure syndromes that can present in adulthood are telomere biology disorders (TBDs). Telomere length testing should, therefore, be performed in patients with cytopenia and suggestive clinical features [9]. In asymptomatic patients with low neutrophil counts who originate from areas with a high prevalence of Duffy-null-associated neutrophil count (DANC) (i.e., Sub-Saharan Africans and Arabs), RBC phenotyping should be performed to identify or rule out the Fy(a-b-) phenotype. DANC is also referred to as ACKR1/DARC-associated neutropenia (ADAN).

The urgency for carrying out a bone marrow examination is routinely guided by the probable cause and trajectory of the neutrophil count, the clinical course, and the urgency for management. Bone marrow examination should generally be considered for patients with unexplained neutropenia. According to the current guideline recommendations [2], omission of bone marrow examination may be acceptable in cases with chronic but mild neutropenia that remains stable over time. The examination should, however, ideally be performed in all patients before initiating granulocyte colony-stimulating factor (G-CSF) treatment. The main objective of a bone marrow investigation is to exclude hematological neoplasms, especially before administration of G-CSF [2]. In cases with additional unexplained cytopenia, unexplained dysplastic features or blasts in the peripheral blood (red flags), a bone marrow examination should be expedited. The same applies for patients with suspected drug or postinfectious acute neutropenia that does not recover after the offending substance or pathogen has been eradicated.

Bone marrow evaluation also helps to assess for cellularity, the presence of dysplasia, and the maturation of the cell lines [10]. Some myeloid line patterns are particular to some entities, and it is, therefore, important to highlight them.

Drug-induced neutropenia may result in different bone marrow patterns, depending on the mechanism of action of the involved substance, its pharmacokinetics, and the time point of the bone marrow examination after drug exposure. Bone marrow examination performed soon after or during drug exposure usually demonstrates a complete or near-complete absence of mature myeloid cells. During early recovery, on the other hand, maturing forms up to a certain stage may be observed.

Patients with severe congenital neutropenia usually show “maturation arrest” with an abundant number of promyelocytes and only few mature myeloid cells [2,10]. At the other extreme, myelokathexis (a feature of inherited WHIM syndrome (warts, hypogammaglobulinemia, infections, and myelokathexis)) is characterized by the presence of unusual abundant mature neutrophils in bone marrow. This is due to difficulties around the cells exiting the marrow and entering the blood [10]. In T-LGLL, bone marrow may show a characteristic pattern of myeloid maturation arrest and increased LGL infiltrates.

Bone marrow evaluation may, however, be normal in patients with chronic idiopathic or autoimmune neutropenia [3,4,11].

As there is an overlap in the presentation of the different causes in bone marrow morphology, this examination is usually not diagnostic when performed as an isolated test. Instead, it is important to interpret bone marrow results in the context of the clinical disease course and the dynamics of neutrophil counts.

It is also important to distinguish disorders of neutrophil dysfunction, such as Chediak–Higashi syndrome, leukocyte adhesion deficiencies, and chronic granulomatous disease, from other forms chronic neutropenia. In these conditions, the neutrophil count may be reduced or normal, but cells may have impaired responses that are critical for host defense; the affected individuals may present with recurrent infections [12].

**Figure 1:**
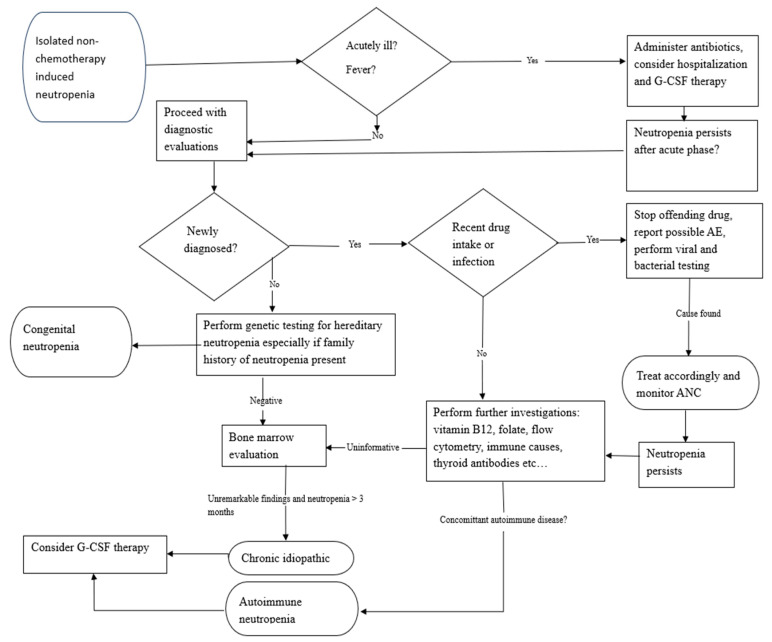
Flow diagram for the diagnostic assessment and management of isolated neutropenia. Abbreviations: AE (adverse event), ANC (absolute neutrophil count), G-CSF (granulocyte colony-stimulating factor). Adapted from ref. [3].

Figure 1 below illustrates the recommended steps to consider for the evaluation and management of adult patients who present with isolated neutropenia.

## 3. Etiologies of Chronic Neutropenia

### 3.1. Drug-Induced Chronic Neutropenia

Drugs and toxins are the most frequent causes of isolated neutropenia in adults [3,5,13]. However, the vast majority of drug-induced neutropenia cases are acute and transient. The following are the drugs most commonly reported to be associated with neutropenia: clozapine, dapsone, dipyrone, pantoprazole, ibuprofen, olanzapine, valaciclovir, thiamazole, mycophenolic acid, levamisole, penicillin G, procainamide, propylthiouracil, rituximab, sulfasalazine, and ticlopidine [4,14].

Drug-induced neutropenia can nevertheless persist for more than 3 months, and, in certain cases, may fail to recover at all or show an incomplete recovery, hence the importance of mentioning it.

Late-onset neutropenia is a rare but potentially severe adverse event, mainly described in association with rituximab, an anti-CD20 antibody, whereby neutropenia develops 4 weeks or more after drug administration. The duration of neutropenia has been reported to last up to 5.2 months [15]. Late-onset neutropenia has also been reported under other monoclonal anti-CD20 antibodies like ocrelizumab and ofatumumab [16], although with shorter ANC recovery times. This calls to attention the importance of frequently monitoring neutrophil counts under B-cell-depleting treatment, as well as accurately reporting drug-associated neutropenia. The successful drug re-challenging of clozapine after an appropriate risk–benefit analysis has been reported in the literature [17]; these strategies should only be attempted if absolutely clinically indicated and with stringent monitoring of blood counts.

### 3.2. Autoimmune Neutropenia (AIN)

In the adult population, primary AIN is relatively rare and is typically associated with a mild clinical course [11]. It is more common in females [4,11,18].

Secondary AIN may develop in the presence of autoimmune diseases—for example, systemic lupus erythematosus, rheumatoid arthritis, or lymphoid malignancies [11]—and is mainly caused by antibody-mediated destruction of neutrophils (Table 2). Felty’s syndrome (a triad of neutropenia, splenomegaly, and rheumatoid arthritis) [19] may also be associated with chronic neutropenia.

As mentioned above, testing for antineutrophil antibodies may aid in diagnosis, but management decisions should not be based on these results alone [2,8].

Therapy of AIN is founded on accurate diagnosis and managing the primary autoimmune disorder. Primary immunodeficiencies, such as common variable immunodeficiency (CVID), have also been associated with secondary neutropenia [11,20]. Common variable immunodeficiency-12 (CVID12), an autosomal dominant subtype of CVID, is an immune condition resulting from loss-of-function mutations of NFKB1 [21,22]. These disorders are characterized by hypogammaglobulinemia and recurrent infections, and their management strategies are different from other autoimmune neutropenias, with immune globulin therapy rather than G-CSF representing the mainstay of management [23]. Correct diagnostic workup is therefore paramount, as this is crucial for therapy decisions.

### 3.3. Chronic Idiopathic Neutropenia (CIN)

CIN is described as neutropenia lasting ≥ 3 months with no identifiable cause. This refers to persistent neutropenia that is not associated with drugs, malignant disease, infections, or immune causes [18] (Table 2). There is an overlap between the terminologies CIN and idiopathic cytopenia of undetermined significance (ICUS), as this term also describes persistent cytopenia(s) without evidence of any underlying hematological disease [24]. The pathogenesis of CIN is linked to excessive production of inflammatory cytokines (such as Fas-ligand and IFNγ) by activated T-lymphocytes that cause apoptosis of myelopoiesis [24,25]. Recently, Papadaki et al. showed that CIN patients have significantly lower proportions of myeloid-derived suppressor cells compared to healthy controls, which probably result in the inadequate suppression of the aberrant inflammatory processes that underlie CIN [26].

The diagnosis of CIN remains a diagnosis of exclusion. A female predominance of CIN has been described in the literature [3,18], with a median age of 28 years [18]. There may be a difference in clinical phenotypes in regard to risk of infections and need for individualized therapy consideration, depending on the clinical course and not on the neutrophil count alone [27].

### 3.4. Clonal Cytopenia of Undetermined Significance (CCUS)

The term CCUS refers to unexplained cytopenia(s) in the presence of somatic mutations of myeloid neoplasm-associated genes at a variant allele fraction of ≥2% without the diagnosis of a hematologic disorder [1] (Table 2). Recognizing this entity is important, as CCUS, unlike CIN, is associated with an increased risk of progression to myelodysplastic neoplasms. This highlights the importance of performing a bone marrow investigation including NGS testing to rule out CCUS in patients with persistent neutropenia.

### 3.5. Duffy-Null Associated Neutrophil Count (DANC)

This condition, previously known as benign ethnic neutropenia, is a hereditary cause of an ANC < 1.5 × 10^9^/L, with no clinical symptoms, that is most often seen in sub-Saharan Africans (80–100%), Arabs (50–70%), and very rarely in people with European or Asian ancestry (<1%) [28,29]. It is correlated with the homozygosity of a single nucleotide polymorphism in the atypical chemokine receptor-1 (*ACKR1*) gene, which is also referred to as the Duffy antigen receptor for chemokines (DARC) [30]; this causes an RBC antigen phenotype termed Duffy-null or Fy(a-b-). The condition is also referred to as *ACKR1/DARC*-associated neutropenia (ADAN) [31,32]. A proposed theory of how the Duffy-null phenotype leads to a lower neutrophil count is that the disruption of the normal inflammatory cytokine domain impairs white blood cell migration and trafficking (as the Duffy RBC antigen binds to and incorporates chemokines into the red blood cell) [33]. The Fy(a-b-) RBC phenotype offers some protection against malaria infection, and is, therefore, an advantageous trait [29].

In regard to diagnostic work-up, detection of homozygosity for ACKR1 rs2814778 or the Duffy-null [Fy(a-b-)] phenotype supports the diagnosis of DANC.

It should also be noted that individuals of African descent have a white blood cell count that is typically 700 cells/μL lower than Caucasian individuals [34]. Laboratories and clinicians should therefore be encouraged to use ancestry-aware laboratory reference ranges to avoid unnecessary costly workup and to prevent healthcare inequities (such as withholding chemotherapy due to perceived neutropenia). No therapy or further diagnostic work-up is needed.

### 3.6. Familial Neutropenia

Familial neutropenia is defined as mild neutropenia in families from ethnicities that are not usually linked with the DANC, although the two entities are phenotypically similar. The condition is usually inherited, although sporadic cases have also been described [35,36,37]. Polymorphisms that may be associated with lower ANCs in familial neutropenia include the lead SNP rs9131 on the *CXCL2* gene, as well as rare variants in *TCIRG1* [38,39]. Neutropenia is generally mild and asymptomatic.

### 3.7. Severe Congenital Neutropenia (SCN)

SCN is a rare form of hereditary neutropenia that presents in infancy with severe neutropenia and often monocytosis. The primary clinical feature of SCN is a high risk of severe bacterial and oral infections.

SCN is associated with mutations in many genes, including *ELANE*, *HAX1*, *G6PC3*, *VPS45*, *WAS*, *CXCR4*, and *GSF3R* [10]. Among them, *ELANE*, which encodes neutrophil elastase, is the most common gene mutation that causes SCN and cyclic neutropenia (CyN) [40,41]. Kostmann Syndrome (*HAX1* mutation) is one subtype of SCN that is frequently accompanied by neurological involvement.

G-CSF is currently the treatment of choice and has, since its availability in 1987, dramatically changed the natural history of the disease, with a significant reduction in the severity and frequency of infections and improved survival [42,43,44]. Allogeneic hematopoietic cell transplantation (allo-HCT) is the only curative option for these patients, and was, before the availability of G-CSF, the only chance of survival [45]. SCN is, furthermore, a pre-malignant condition, with predisposition to clonal hematopoietic diseases such as acute myeloid leukemia (AML) and myelodysplastic neoplasms (MDS) [46]. The major drivers of the evolution of secondary malignancies are acquired mutations in the *CSF3R* gene and *RUNX1* genes [46]. Loss-of-function GATA2 mutations have also been identified as a cause of congenital neutropenia associated with a high risk of leukemic transformation [46,47].

Allo-HCT should still be considered for those patients who are refractory to G-CSF therapy, those who have a higher risk profile of transformation, or those who undergo malignant transformation [45,46].

Recently, CRISPR-Cas9n-mediated gene editing to correct ELANE mutations in patient-derived hematopoietic stem cells has been explored as a potentially safe, efficient, and curative gene therapy approach for ELANE-SCN patients [48]. This approach, however, currently remains investigational.

Frequent evaluations to monitor the clinical course and detect possible chromosomal abnormalities are, therefore, recommended. SCN patients who respond well to G-CSF therapy can be followed every 3 months. Since chemotherapy is relatively inefficient in AML which develops from SCN, an annual bone marrow examination for early detection of myeloid malignancy is advised [5,47].

### 3.8. Cyclic Neutropenia (CyN)

CyN is an inherited disease of recurrent fluctuating neutropenia, usually every 21 days, that results from mutations in the *ELANE* gene; these mutations are consistently found in all patients suffering from CyN [5,49]. Unlike SCN, CyN patients tend to have a mild clinical course and can, therefore, be diagnosed in adulthood. Patients may present with fever, malaise, mucosal ulcerations, and other infections during neutrophil nadir. Diagnostic work-up entails blood counts two times a week for approximately 4–6 weeks to demonstrate the cyclic or periodic pattern of neutropenia. CyN is not associated with an increased risk of evolution to AML.

### 3.9. Congenital Conditions Associated with Neutropenia

A number of congenital syndromes may present with chronic neutropenia; examples of these are Shwachman–Diamond syndrome, WHIM syndrome, Fanconi anemia, and telomere biology disorders (TBDs) [5,9,10]. The presence of unexplained persistent neutropenia in combination with somatic findings (e.g., pancreatic dysfunction, premature graying of the hair, abnormalities in the fingernails or skeleton), or a family history of neutropenia should prompt an investigation for congenital forms of neutropenia, even in adult patients. The importance of genetic testing ought to be emphasized as part of the diagnostic work-up, and clinicians should be educated about these techniques and their capabilities to correctly characterize these patients.

Although congenital forms of neutropenia are usually diagnosed in early childhood, adult diagnosis can occur, especially in mild, cryptic, or atypical cases. In a review of the literature published by Alter P., 13% of SCN cases, 9% of Fanconi anemia cases, and 46% of patients with Dyskeratosis congenita were diagnosed ≥ 16 y of age [50]. One case report describes a case of *ELANE*-SCN diagnosed at the age of 29 years [51].

As the prognosis of SCN has dramatically improved in recent years, these patients are now surviving into adulthood. It is, therefore, imperative that adult hematologists be aware of these conditions and their management.

## 4. Treatment of Chronic Neutropenia

Treatment of chronic neutropenia should be based on the etiology, severity of neutropenia, and, most importantly, patient-specific factors such as history of recurrent infectious complications [2,7,52]. Although the risk of infections is, in most cases, determined by the severity and duration of neutropenia and bone marrow reserve, certain subtypes of chronic neutropenia are generally associated with mild disease; these include CIN, AIN, and CyN. SCN, on the other hand, is associated with severe disease. Drug-induced neutropenia, depending on the degree of neutropenia, could also lead to severe disease. Individuals with DANC, as already mentioned, are asymptomatic.

G-CSF is a drug in the class of human cytokines that triggers the growth and differentiation of myeloid precursors and boosts their maturation into neutrophils [46]. An increase in neutrophil values is usually seen within 24–48 h after drug initiation.

Ideally, the lowest effective dose of G-CSF should be used for infection control; an ANC  ≥  1.0  ×  10^9^/L is usually considered the protective threshold against infections [7].

As mentioned above, SCN patients usually need life-time G-CSF treatment to attain therapeutic neutrophil counts ≥ 1.0 × 10^9^/L and ≤5.0 × 10^9^/L [44]. A starting dose of 5 mcg/kg/day is recommended, with dose escalation according to response [7,42,53].

For chronic neutropenia patients other than SCN, an individual approach based on the frequency and gravity of infections rather than the neutrophil count alone is recommended [7,18]. Most patients may only require on-demand G-CSF therapy, for instance, before surgery or during infections, but not continuously. A starting dose of 1–5 mcg/kg/day 2–3 times a week is generally sufficient [7] (Table 3).

Common side effects of G-CSF therapy include bone pain, myalgias, headache, skin rash, splenomegaly, thrombocytopenia, decreased bone density, and osteoporosis. Although a possible link between G-CSF administration and hematological neoplasms has been suggested in the literature, long-term studies have shown this therapy to be remarkably safe [48,54]. This is also true in pregnancy: G-CSF in pregnant women with chronic neutropenia has been shown to be safe and effective [3,55,56].

Other treatments for neutropenia—for example, corticosteroids, androgens, splenectomy, or granulocyte transfusions—are not generally recommended [7,18]. Nevertheless, secondary chronic neutropenia associated with systemic disease, as is the case with CVID, may require immune globulin therapy [23]. Treatment for autoimmune neutropenia secondary to rheumatologic disorders, such as systemic lupus erythematosus, similarly may require immunosuppressive therapy for the underlying disease. Prophylactic antibiotic therapy in chronic neutropenia is not recommended [18].

It should not be forgotten that fever in neutropenia is a medical emergency which could be life-threatening and requires prompt treatment with broad-spectrum empiric antibiotics. Old age, poor performance status, septicemia, renal failure, ANC ≤ 0.1 × 10^9^/L, duration of neutropenia, procalcitonin levels, and low MASCC (Multinational Association for Supportive Care in Cancer risk-index score) for chemotherapy-induced neutropenia are consensually accepted as poor prognostic factors [6,13] and usually require hospital admission for intravenous broad-spectrum antibiotics and G-CSF.

Additionally, routine vaccines against bacterial and viral diseases are indicated for patients with chronic neutropenia without any additional immune defect, according to the respective national regulations [7].

## 5. Conclusions

In conclusion, physicians should be aware that chronic isolated neutropenia is rare and its etiologies are heterogeneous. They should also be cognizant of the steps to consider in its diagnostic assessment and management.

Chronic neutropenia can be inherited or acquired. Inherited forms, even in adult patients, ought to still be considered in cases with unexplained chronic neutropenia.

Lastly, therapy considerations should be based on the etiology and severity of neutropenia, as well as the clinical course.

## Figures and Tables

**Table 1:** Causes of acute versus chronic isolated neutropenia.

Acute Neutropenia (Duration < 3 Months)	Chronic Neutropenia (Duration > 3 Months)
Drugs/toxins	Severe congenital neutropenia
Infections	Cyclic neutropenia
Autoimmune causes	Chronic idiopathic neutropenia
Nutritional deficiencies	Autoimmune neutropenia
Radiotherapy	Duffy-null associated neutrophil count
Hematological diseases, e.g., AA, AML	Rarely drugs, e.g., rituximab
Rheumatological disorders	Hematological diseases, e.g., LGLL, SDS, TBD

Abbreviations: AA (aplastic anemia), AML (acute myeloid leukemia), LGLL (Large Granular Lymphocytic Leukemia), SDS (Shwachman–Diamond syndrome), TBD (Telomere Disorders).

**Table 2:** Key differences between CIN, AIN, and CCUS.

	CIN	AIN	CCUS
**Cause**	No identifiable cause	Usually develops in the presence of autoimmune diseases	Somatic mutations of myeloid neoplasm-linked genes
**Clonal evolution**	Not associated with transformation to myeloid malignancy	Not associated with transformation to myeloid malignancy	Increased risk of progression to myelodysplastic neoplasms
**Diagnosis**	Exclusion of other causes of neutropenia (drugs, malignant disease, infections, nutritional deficiencies, or autoimmune causes)	Detection of anti-neutrophil antibodies possible	NGS myeloid panel showing somatic mutations
**Clinical course**	Generally benign, with mild symptoms/infections	Generally benign with mild symptoms/infections; may be acute or chronic	May be asymptomatic; transformation to myelodysplastic neoplasms is possible
**Bone marrow examination**	Typically normal, without clonal abnormalities	Typically normal, without clonal abnormalities	Typically normal or mild dysplasia
**Therapy**	G-CSF either on-demand or continuously (in patients with frequent infections)	Treatment of underlying autoimmune disease; G-CSF either on-demand or continuously (in patients with frequent infections)	Monitoring; treatment, if progression occurs

Abbreviations: CIN (Chronic idiopathic neutropenia), AIN (Autoimmune neutropenia), CCUS (Clonal cytopenia of undetermined significance), VAF (variant allele fraction) [1,11,18].

**Table 3:** Recommended G-CSF dosing strategies.

SCN	Starting dose 5 mcg/kg/day with dose increment by 1–2 mcg/kg/day weekly until target ANC ≥ 1.0 × 10^9^/L is achieved (median dose 7.3 mcg/kg/day)
CyN	Median dose 2.5 mcg/kg/d
CIN/AIN	Median dose 1.2 mcg/kg/day

G-CSF can be given daily, every 2 days or 2–3 times per week depending on response. Abbreviations: SCN (Severe congenital neutropenia), ANC (absolute neutrophil count), CyN (Cyclic neutropenia), CIN (Chronic idiopathic neutropenia), AIN (Autoimmune neutropenia) [7,11,53].

## Data Availability

No new data were created or analyzed in this study.

## References

[1] Khoury J.D., Solary E., Abla O., Akkari Y., Alaggio R., Apperley J.F., Bejar R., Berti E., Busque L., Chan J.K.C. (2022). The 5th edition of the World Health Organization Classification of Haematolymphoid Tumours: Myeloid and Histiocytic/Dendritic Neoplasms. Leukemia.

[2] Fioredda F., Skokowa J., Tamary H., Spanoudakis M., Farruggia P., Almeida A., Guardo D., Hoglund P., Newburger P.E., Palmblad J. (2023). The European Guidelines on Diagnosis and Management of Neutropenia in Adults and Children: A Consensus Between the European Hematology Association and the EuNet-INNOCHRON COST Action. Hemasphere.

[3] Njue L., Porret N., Schnegg-Kaufmann A.S., Varra L.F., Andres M., Rovo A. (2024). Isolated Severe Neutropenia in Adults, Evaluation of Underlying Causes and Outcomes, Real-World Data Collected over a 5-Year Period in a Tertiary Referral Hospital. Medicina.

[4] Rout P., Reynolds S.B., Zito P.M. (2025). Neutropenia.

[5] Min K.I., Byeon S. (2025). Diagnosis and management of neutropenia. Blood Res..

[6] Sandherr M., Stemler J., Schalk E., Hattenhauer T., Hentrich M., Hertenstein B., Hohmann C., Mellinghoff S.C., Mispelbaum R., Rieger C. (2025). 2024 update of the AGIHO guideline on diagnosis and empirical treatment of fever of unknown origin (FUO) in adult neutropenic patients with solid tumours and hematological malignancies. Lancet Reg. Health Eur..

[7] Fioredda F., Spanoudakis M., Skokowa J., Tamary H., Farruggia P., Almeida A., Guardo D., Palmblad J., Hoglund P., Touw I.P. (2025). European guidelines on treatment and supportive measures in chronic neutropenias: A consensus between the European Hematology Association and the EuNet-INNOCHRON COST Action based on a systematic evidence review. Hemasphere.

[8] Karakilic-Ozturan E., Karaman S., Soguksu P., Mese S., Agacfidan A., Mutlu U.D., Karakas Z., Tugcu D., Karagenc-Ozkan A., Devecioglu O. (2020). The Role of Anti-Neutrophil Antibodies in the Etiologic Classification of Childhood Neutropenia: A Cross-Sectional Study in a Tertiary Center. J. Pediatr. Hematol. Oncol..

[9] Kam M.L.W., Nguyen T.T.T., Ngeow J.Y.Y. (2021). Telomere biology disorders. npj Genom. Med..

[10] Donadieu J., Beaupain B., Fenneteau O., Bellanne-Chantelot C. (2017). Congenital neutropenia in the era of genomics: Classification, diagnosis, and natural history. Br. J. Haematol..

[11] Fioredda F., Dufour C., Hoglund P., Papadaki H.A., Palmblad J. (2023). Autoimmune Neutropenias: Update on Clinical and Biological Features in Children and Adults. Hemasphere.

[12] Dinauer M.C. (2020). Neutrophil Defects and Diagnosis Disorders of Neutrophil Function: An Overview. Methods Mol. Biol..

[13] Andres E., Mourot-Cottet R. (2017). Non-chemotherapy drug-induced neutropenia—An update. Expert Opin. Drug Saf..

[14] Wu S., Huang L., Chen J., Xie X., Huang S., Huang X. (2025). Non-chemotherapy drugs inducing agranulocytosis: A disproportionality analysis based on the FAERS database. Front. Pharmacol..

[15] Malpica Castillo L.E., Palmer S., Zhu A., Deal A.M., Chen S.L., Moll S. (2020). Incidence and time course of neutropenia in patients treated with rituximab-based therapy for non-malignant immune-mediated hematologic diseases. Am. J. Hematol..

[16] Protopapa M., Schraad M., Pape K., Steffen F., Steenken L., Zipp F., Fleischer V., Bittner S. (2025). Recurrent late-onset neutropenia following treatment with different B cell-depleting strategies in multiple sclerosis. Med.

[17] Silva E., Higgins M., Hammer B., Stephenson P. (2020). Clozapine rechallenge and initiation despite neutropenia—A practical, step-by-step guide. BMC Psychiatry.

[18] Dale D.C., Bolyard A.A. (2017). An update on the diagnosis and treatment of chronic idiopathic neutropenia. Curr. Opin. Hematol..

[19] Wegscheider C., Ferincz V., Schols K., Maieron A. (2023). Felty’s syndrome. Front. Med..

[20] Cunningham-Rundles C., Casanova J.L., Boisson B. (2024). Common variable immunodeficiency: Autoimmune cytopenias and advances in molecular diagnosis. Hematol. Am. Soc. Hematol. Educ. Program..

[21] Fliegauf M., Kruger R., Steiner S., Hanitsch L.G., Buchel S., Wahn V., von Bernuth H., Grimbacher B. (2021). A Pathogenic Missense Variant in NFKB1 Causes Common Variable Immunodeficiency Due to Detrimental Protein Damage. Front. Immunol..

[22] Yin J., Hayes K.M., Ong M.S., Mizgerd J.P., Cunningham-Rundles C., Dominguez I., Barmettler S., Farmer J.R., Maglione P.J. (2025). Common Variable Immunodeficiency Clinical Manifestations Are Shaped by Presence and Type of Heterozygous NFKB1 Variants. J. Allergy Clin. Immunol. Pract..

[23] Yazdani R., Habibi S., Sharifi L., Azizi G., Abolhassani H., Olbrich P., Aghamohammadi A. (2020). Common Variable Immunodeficiency: Epidemiology, Pathogenesis, Clinical Manifestations, Diagnosis, Classification, and Management. J. Investig. Allergol. Clin. Immunol..

[24] Tsaknakis G., Galli A., Papadakis S., Kanellou P., Elena C., Todisco G., Bono E., Rizzo E., Molteni E., Fragiadaki I. (2021). Incidence and prognosis of clonal hematopoiesis in patients with chronic idiopathic neutropenia. Blood.

[25] Spanoudakis M., Koutala H., Ximeri M., Pyrovolaki K., Stamatopoulos K., Papadaki H.A. (2010). T-cell receptor Vbeta repertoire analysis in patients with chronic idiopathic neutropenia demonstrates the presence of aberrant T-cell expansions. Clin. Immunol..

[26] Bizymi N., Damianaki A., Aresti N., Karasachinidis A., Vlata Z., Lavigne M., Dialynas E., Gounalaki N., Stratidaki I., Tsaknakis G. (2024). Characterization of myeloid-derived suppressor cells in the peripheral blood and bone marrow of patients with chronic idiopathic neutropenia. Hemasphere.

[27] Fattizzo B., Bosi A., Sorrenti M., Murgia D., Pettine L., Bortolotti M., Croci G.A., Passamonti F., Barcellini W. (2024). Natural history of chronic idiopathic neutropenia of the adult. Sci. Rep..

[28] Atallah-Yunes S.A., Ready A., Newburger P.E. (2019). Benign ethnic neutropenia. Blood Rev..

[29] Merz L.E., Achebe M. (2021). When non-Whiteness becomes a condition. Blood.

[30] Reich D., Nalls M.A., Kao W.H., Akylbekova E.L., Tandon A., Patterson N., Mullikin J., Hsueh W.C., Cheng C.Y., Coresh J. (2009). Reduced neutrophil count in people of African descent is due to a regulatory variant in the Duffy antigen receptor for chemokines gene. PLoS Genet..

[31] Liu J.M., Luo H.R. (2024). Novel neutrophil biology insights underlying atypical chemokine receptor-1/Duffy antigen receptor of chemokines-associated neutropenia. Curr. Opin. Hematol..

[32] Palmblad J., Sohlberg E., Nilsson C.C., Lindqvist H., Deneberg S., Ratcliffe P., Meinke S., Mortberg A., Klimkowska M., Hoglund P. (2024). Clinical and immunological features in ACKR1/DARC-associated neutropenia. Blood Adv..

[33] Pruenster M., Mudde L., Bombosi P., Dimitrova S., Zsak M., Middleton J., Richmond A., Graham G.J., Segerer S., Nibbs R.J. (2009). The Duffy antigen receptor for chemokines transports chemokines and supports their promigratory activity. Nat. Immunol..

[34] Lim E., Miyamura J., Chen J.J. (2015). Racial/Ethnic-Specific Reference Intervals for Common Laboratory Tests: A Comparison among Asians, Blacks, Hispanics, and White. Hawaii J. Med. Public Health.

[35] Solomou E.E., Salamaliki C., Lagadinou M. (2021). How to Make the Right Diagnosis in Neutropenia. Clin. Hematol. Int..

[36] Busch F.H. (1990). Familial benign chronic neutropenia in a Danish family. Ugeskr. Laeger.

[37] Denic S., Showqi S., Klein C., Takala M., Nagelkerke N., Agarwal M.M. (2009). Prevalence, phenotype and inheritance of benign neutropenia in Arabs. BMC Blood Disord..

[38] Reiner A.P., Lettre G., Nalls M.A., Ganesh S.K., Mathias R., Austin M.A., Dean E., Arepalli S., Britton A., Chen Z. (2011). Genome-wide association study of white blood cell count in 16,388 African Americans: The continental origins and genetic epidemiology network (COGENT). PLoS Genet..

[39] Rosenthal E.A., Makaryan V., Burt A.A., Crosslin D.R., Kim D.S., Smith J.D., Nickerson D.A., Reiner A.P., Rich S.S., Jackson R.D. (2016). Association Between Absolute Neutrophil Count and Variation at TCIRG1: The NHLBI Exome Sequencing Project. Genet. Epidemiol..

[40] Rydzynska Z., Pawlik B., Krzyzanowski D., Mlynarski W., Madzio J. (2021). Neutrophil Elastase Defects in Congenital Neutropenia. Front. Immunol..

[41] Arun A.K., Senthamizhselvi A., Hemamalini S., Edison E.S., Korula A., Fouzia N.A., George B., Mathews V., Balasubramanian P. (2018). Spectrum of ELANE mutations in congenital neutropenia: A single-centre study in patients of Indian origin. J. Clin. Pathol..

[42] Dale D.C., Cottle T.E., Fier C.J., Bolyard A.A., Bonilla M.A., Boxer L.A., Cham B., Freedman M.H., Kannourakis G., Kinsey S.E. (2003). Severe chronic neutropenia: Treatment and follow-up of patients in the Severe Chronic Neutropenia International Registry. Am. J. Hematol..

[43] Welte K., Zeidler C., Dale D.C. (2006). Severe congenital neutropenia. Semin. Hematol..

[44] Dale D.C., Bonilla M.A., Davis M.W., Nakanishi A.M., Hammond W.P., Kurtzberg J., Wang W., Jakubowski A., Winton E., Lalezari P. (1993). A randomized controlled phase III trial of recombinant human granulocyte colony-stimulating factor (filgrastim) for treatment of severe chronic neutropenia. Blood.

[45] Fioredda F., Iacobelli S., van Biezen A., Gaspar B., Ancliff P., Donadieu J., Aljurf M., Peters C., Calvillo M., Matthes-Martin S. (2015). Stem cell transplantation in severe congenital neutropenia: An analysis from the European Society for Blood and Marrow Transplantation. Blood.

[46] Dobrewa W., Bielska M., Babol-Pokora K., Janczar S., Mlynarski W. (2024). Congenital neutropenia: From lab bench to clinic bedside and back. Mutat. Res. Rev. Mutat. Res..

[47] Liu Y.C., Eldomery M.K., Maciaszek J.L., Klco J.M. (2025). Inherited Predispositions to Myeloid Neoplasms: Pathogenesis and Clinical Implications. Annu. Rev. Pathol..

[48] Dale D.C., Bolyard A.A., Shannon J.A., Connelly J.A., Link D.C., Bonilla M.A., Newburger P.E. (2022). Outcomes for patients with severe chronic neutropenia treated with granulocyte colony-stimulating factor. Blood Adv..

[49] Zergham A.S., Acharya U., Mukkamalla S.K.R. (2025). Cyclic Neutropenia.

[50] Alter B.P. (2007). Diagnosis, genetics, and management of inherited bone marrow failure syndromes. Hematol. Am. Soc. Hematol. Educ. Program..

[51] Cerqueira R., Braga J.A.P., Moritz E., Pesquero J.B., Bordin J.O. (2025). A novel ELANE variant causing severe congenital neutropenia diagnosed in adulthood. Blood Cells Mol. Dis..

[52] Long B., Koyfman A. (2025). Incidental neutropenia: An emergency medicine focused approach. Am. J. Emerg. Med..

[53] Dale D.C. (2016). How I diagnose and treat neutropenia. Curr. Opin. Hematol..

[54] Dale D.C., Bolyard A., Marrero T., Makaryan V., Bonilla M., Link D.C., Newburger P., Shimamura A., Boxer L.A., Spiekerman C. (2017). Long-Term Effects of G-CSF Therapy in Cyclic Neutropenia. N. Engl. J. Med..

[55] Zeidler C., Grote U.A., Nickel A., Brand B., Carlsson G., Cortesao E., Dufour C., Duhem C., Notheis G., Papadaki H.A. (2014). Outcome and management of pregnancies in severe chronic neutropenia patients by the European Branch of the Severe Chronic Neutropenia International Registry. Haematologica.

[56] Boxer L.A., Bolyard A.A., Kelley M.L., Marrero T.M., Phan L., Bond J.M., Newburger P.E., Dale D.C. (2015). Use of granulocyte colony-stimulating factor during pregnancy in women with chronic neutropenia. Obstet. Gynecol..

